# Supplementary material for: m6A Modified Short RNA Fragments Inhibit Cytoplasmic TLS/FUS Aggregation Induced by Hyperosmotic Stress
Source: Int J Mol Sci. 2021 Oct 12;22(20):11014. doi: 10.3390/ijms222011014 (PMC8539258; doi:10.3390/ijms222011014)
Supplement: Supplementary file 1 [file ijms-22-11014-s001.zip › ijms-1380301-supplementary.pdf]

Supplementary Materials Figure S1

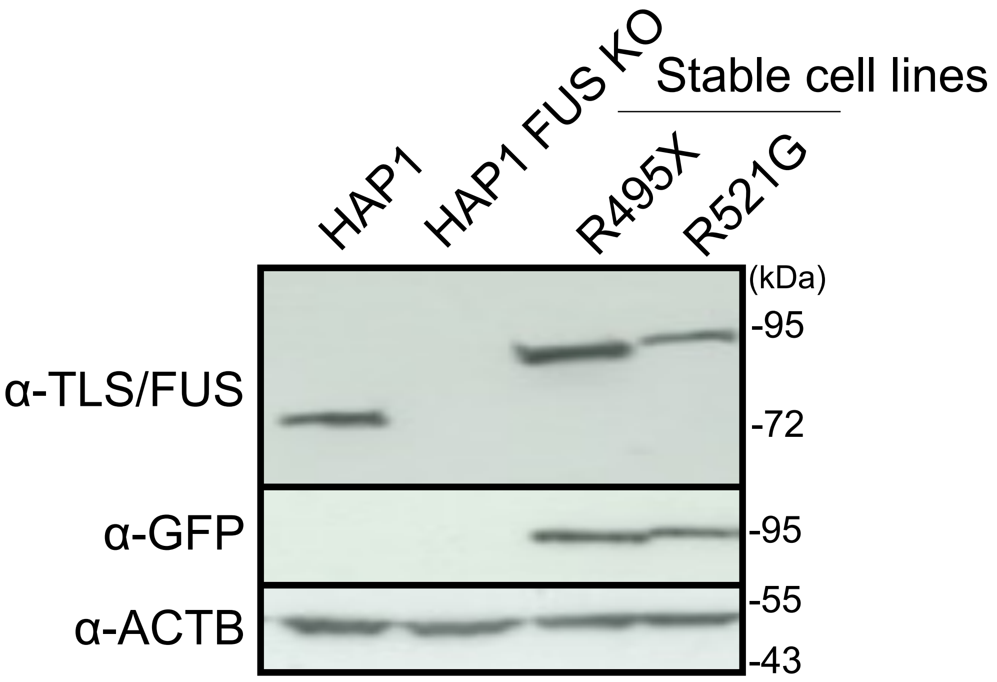

Figure S1. Western blot analysis with whole cell extract of HAP1, HAP1 FUS-KO, and GFP-TLS/FUS mutants expressing stable cell lines. Antibody against TLS/FUS, GFP, and ACTB (as a loading control) was used as primary antibodies.

Supplementary Materials Figure S2

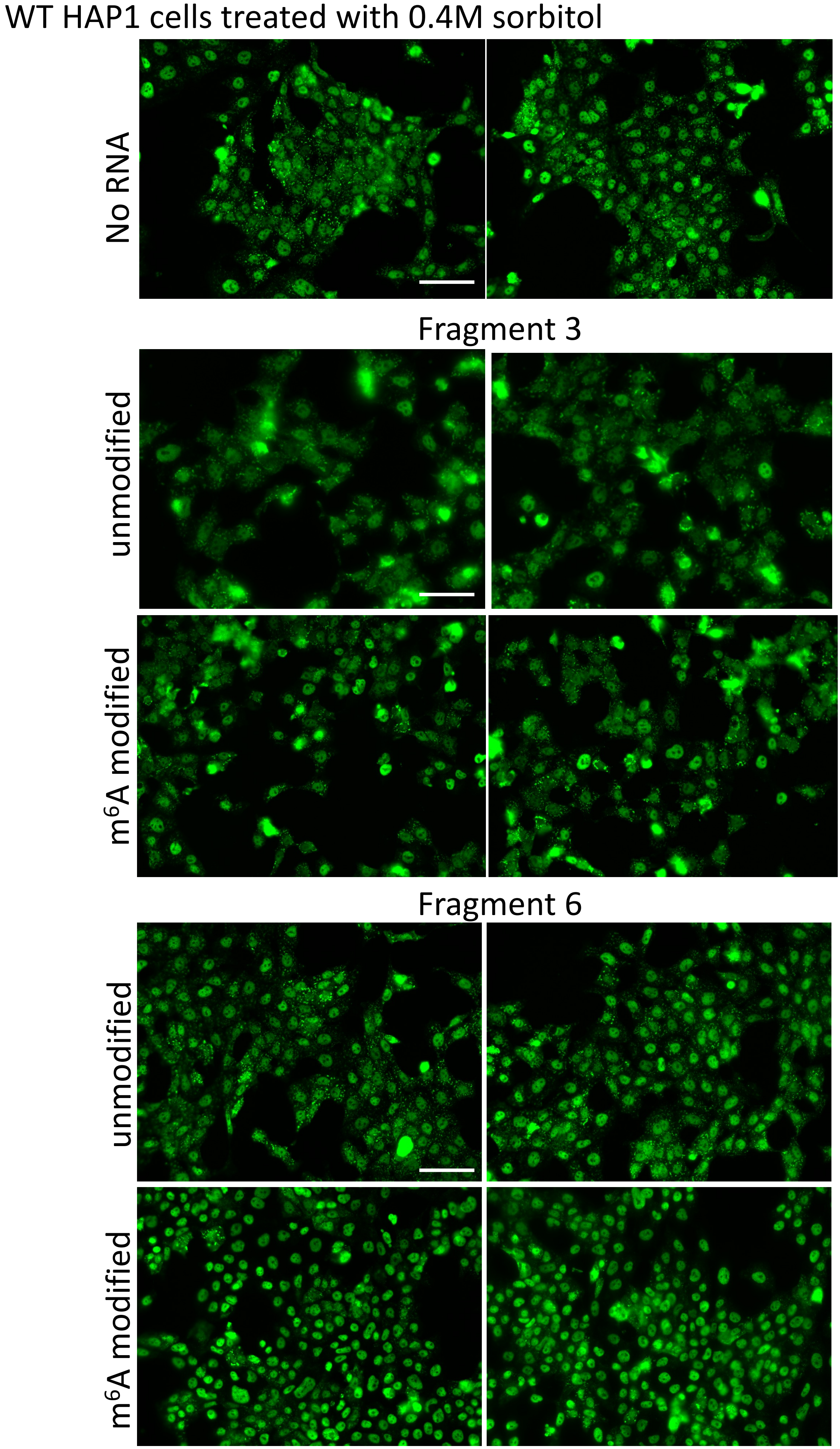

Figure S2. Sample images of ICC detecting TLS/FUS signals of HAP1 cells transfected with indicated RNA fragments prior to 0.4M sorbitol treatment. The images were used for quantification in Figure 3D and 3E. Scale bars = 50 µm.

Supplementary Materials Figure S3

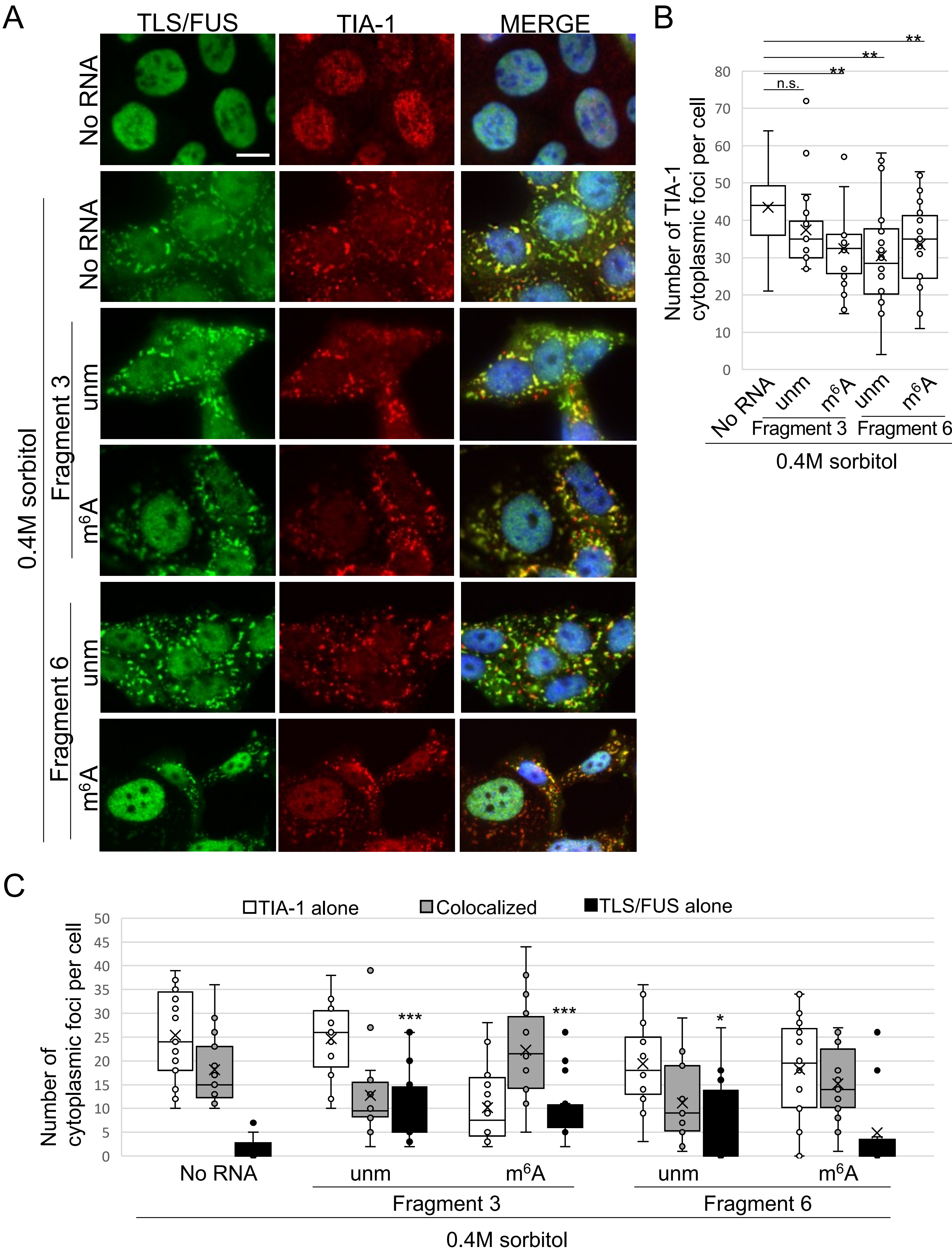

Figure S3. Stress granule formation induced by sorbitol treatment was reduced by transfection of RNA fragments. (A) RNA Fragment 3 or 6 (with or without m^{6}A modification) were transfected to HAP1 cells prior to 0.4M sorbitol treatment. Representative images of ICC are shown. MERGE images indicate the layered images of TLS/FUS, TIA-1 and DAPI (for nuclei staining). Scale bar = 10 μm. (B and C) Cytoplasmic foci in (A) were quantified. n = 20. n.s., not significant; unm, unmodified fragment; *m^{6}A*, m^{6}A modified fragment. *$p < 0.05$, **$p < 0.01$, ***$p < 0.005$.

Supplementary Materials Figure S4

GFP-R521G stable cell line treated with 0.4M sorbitol

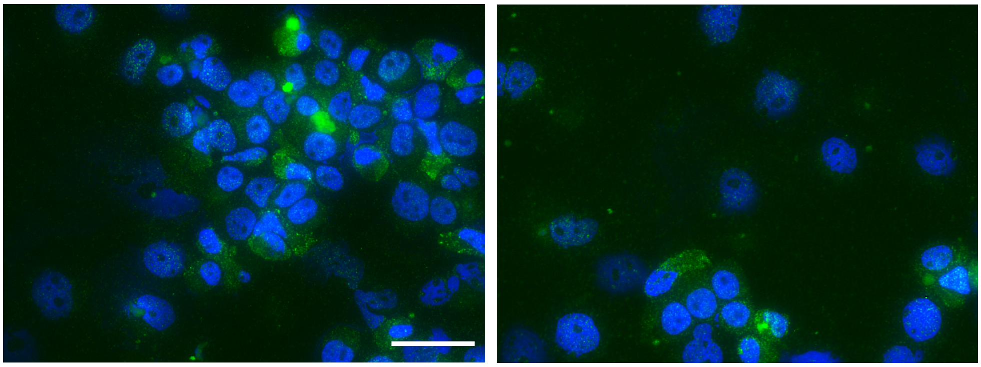

Figure S4. Sample images of GFP signals of stable cell line expressing GFP-R521G treated with 0.4M sorbitol. Scale bar = 30 µm.

Supplementary Materials Figure S5

GFP-R495X stable cell line treated with 0.4M sorbitol

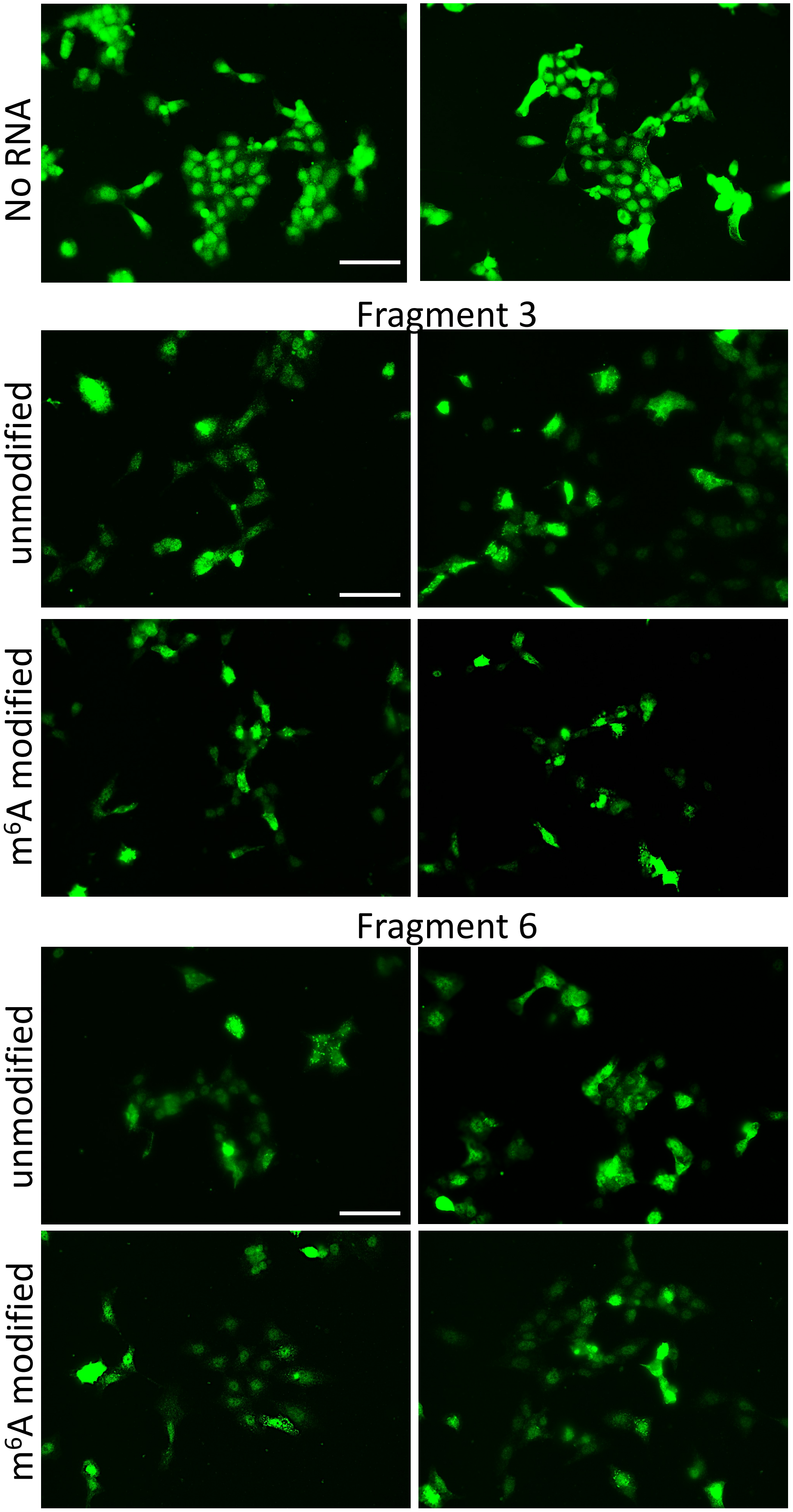

Figure S5. Sample images of GFP signals of stable cell line expressing GFP-R495X transfected with indicated RNA fragments prior to 0.4M sorbitol treatment. The images were used for quantification in Figure 4B and 4C. Scale bars = 50 μm.

Supplementary Materials Figure S6

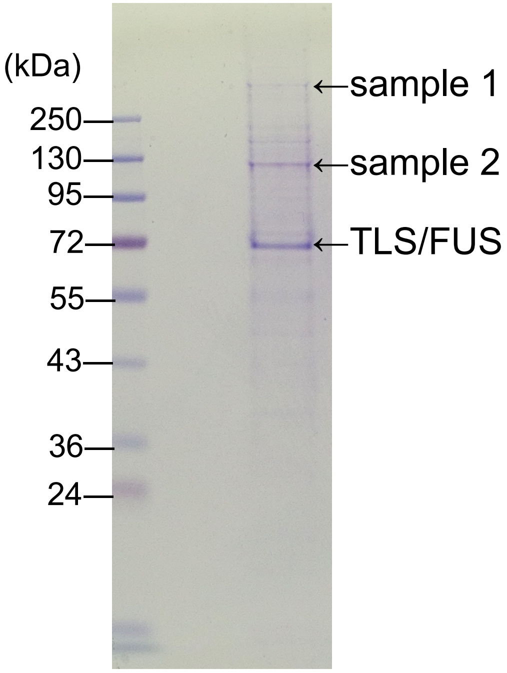

Sample No.		Protein	gene
1	1	YLP motif-containing protein 1 ZAP3	YLPM1<br>C14orf170
	2	Zinc finger protein 638	ZNF638<br>NP220
2	1	Matrin-3	MATR3<br>KIAA0723
	2	Heterogeneous nuclear ribonucleoprotein U	HNRNPU<br>C1orf199
	3	Nucleolar and coiled-body phosphoprotein 1	NOLC1<br>KIAA0035
	4	Interleukin enhancer-binding factor 3	ILF3<br>DRBF

Figure S6. CBB staining after coimmunoprecipitation assay of TLS/FUS antibody incubated with nuclear extract. Bands indicated as sample 1 and 2 were cut out and mass spectrometry analysis predicted the proteins in the right table.

Supplementary Materials Figure S7

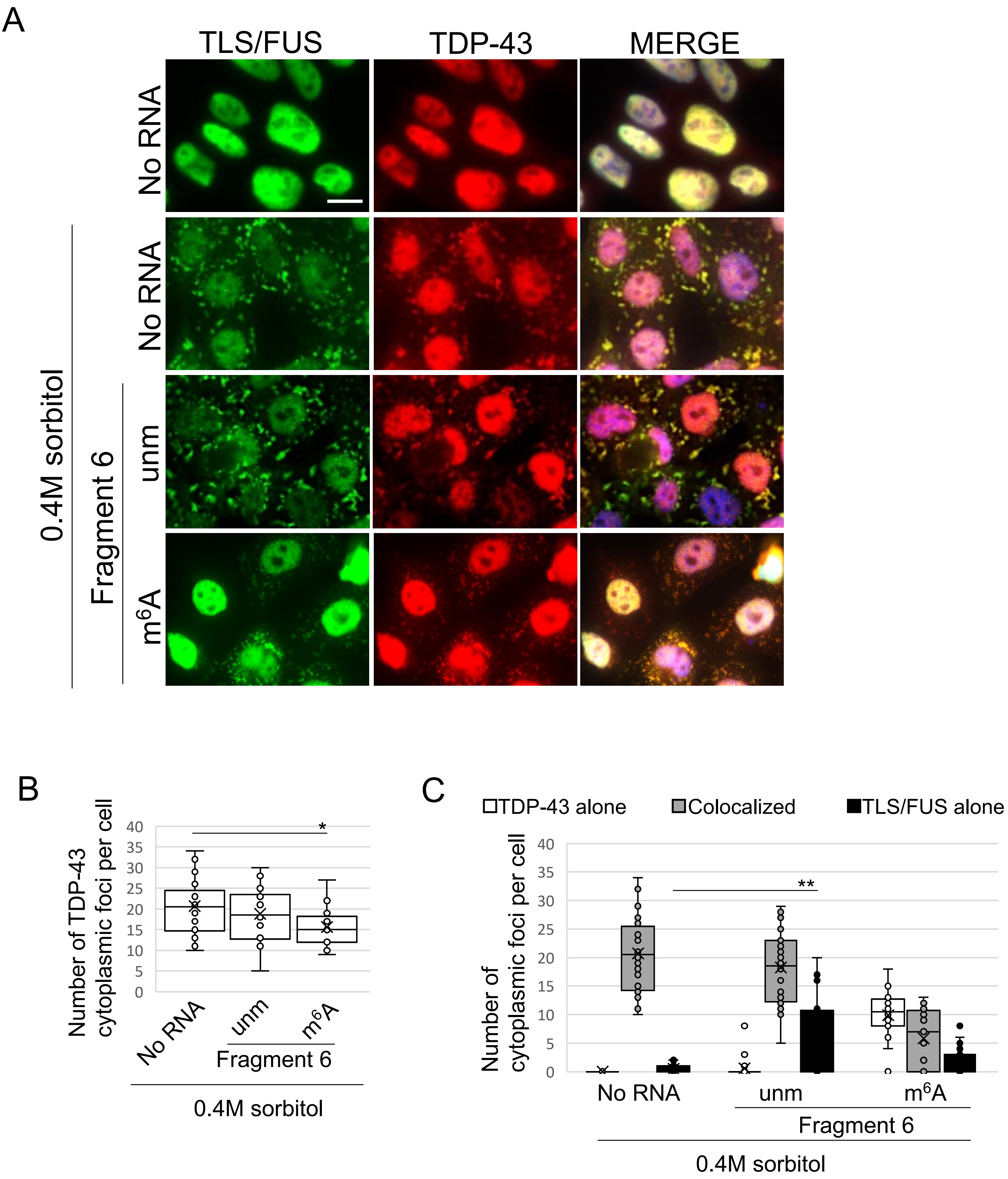

Figure S7. TDP-43 colocalized with cytoplasmic TLS/FUS foci after sorbitol treatment, but m^6A modified RNA fragment impaired this colocalization. RNA Fragment 6 with or without m^6A modification were transfected to HAP1 cells prior to 0.4M sorbitol treatment. Representative images of ICC are shown. MERGE images indicate the layered images of TLS/FUS, Matrin3 and DAPI (for nuclei staining). Scale bar = 10 μm. (B and C) Cytoplasmic foci in (A) were quantified. n = 20. unm, unmodified fragment; m^6A, m^6A modified fragment. *$p < 0.05$, **$p < 0.01$.

Supplementary Materials Figure S8

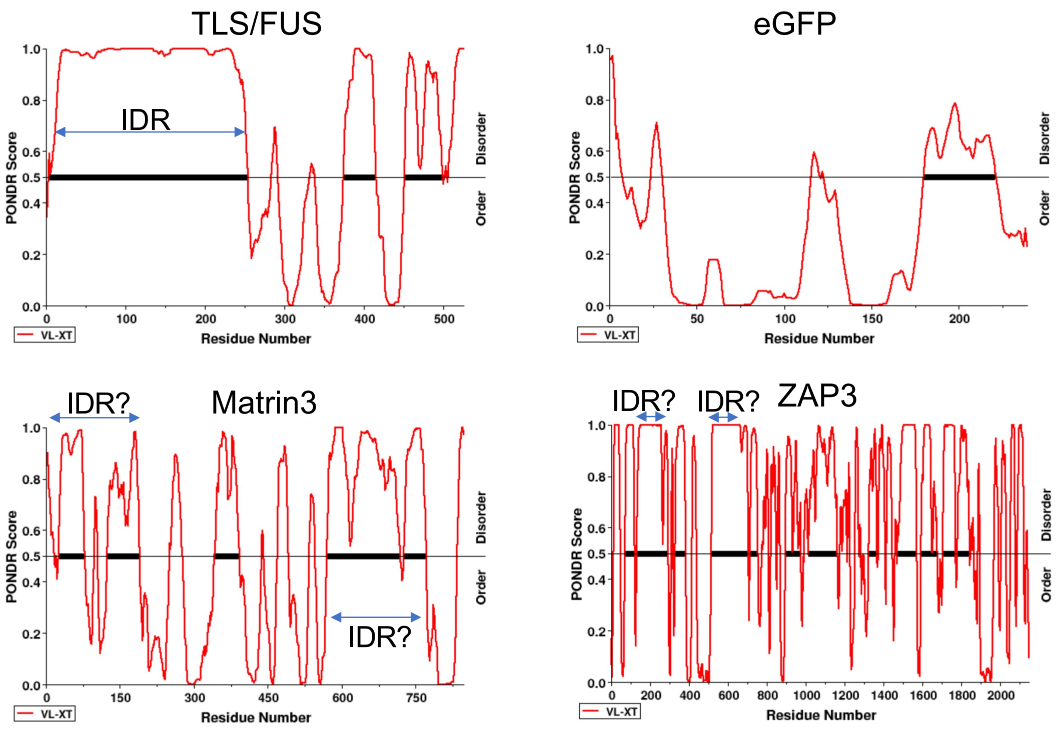

Figure S8. IDRs of TLS/FUS, Matrin3, ZAP3, and eGFP were prediction by PONDR. eGFP was analyzed as a representative structured protein.
